# Knowledge, attitudes, and practices towards dengue prevention among primary school children with and without experience of previous dengue infection in southern Thailand

**DOI:** 10.1016/j.onehlt.2021.100275

**Published:** 2021-06-07

**Authors:** Charuai Suwanbamrung, Bussarawadee Saengsuwan, Thamonwan Sangmanee, Napaporn Thrikaew, Poungpen Srimoung, Sarunya Maneerattanasak

**Affiliations:** a School of Public Health, Walailak University, Nakhon Si Thammarat, Thailand; b Excellent Center for Dengue and Community Public Health (EC for DACH), Walailak University, Nakhon Si Thammarat, Thailand; c Community Public Health Program, School of Public Health, Walailak University, Nakhon Si Thammarat, Thailand; d Maharaj Nakhon Si Thammarat Hospital, Nakhon Si Thammarat Province, Thailand

**Keywords:** Dengue, Knowledge, Attitude, Practice, School children, Thailand

## Abstract

To develop more effective intervention strategies against dengue, it is necessary to identify determinants of knowledge, attitudes, and practices (KAP), which may be influenced by the dengue experiences of the population at risk. The aim of this study was to assess and compare KAP regarding dengue prevention between Thai primary school children with and without experiences of dengue. A cross-sectional study was conducted among children between ages 8 and 13, attending the 50 public primary schools in Kanchanadit district, between October and November 2019. A 32-item questionnaire was used to collect children's socio-demographic characteristics (4 items), health information (2 items), knowledge (10 items), attitudes (7 items), and practices (9 items) towards dengue prevention, which required 30 min to complete. The KAP between groups was then statistically compared, to identify possible causes of observed differences. Of 1979 children, 15.6% self-reported that they had been infected with dengue, while 84.4% had no history of the disease. Most children indicated that they obtained dengue-related information from primary school teachers (73.6%) and their parents (68.5%). No statistically significant differences in mean KAP scores were observed between children with and without dengue experiences (*P* > 0.05). When KAP scores were categorized as good or poor levels, based on an 80% cut-off, 12.3% of all children had good dengue-related knowledge, 41.6% had good attitudes, and 25.9% reported good preventive practices. Dengue experience was significantly and positively associated with exercising good preventive practices (odds ratio [OR] = 1.34, 95% confidence interval [CI]: 1.03–1.75, *P* = 0.031). There were significant positive correlations between attitudes and practices in both children with and without dengue experiences (*P* < 0.001). To enhance KAP towards dengue prevention, further efforts are needed to increase routine dengue health education programs for primary school students who have and have not experienced dengue, and to improve health education programs within communities, especially to assist guardians with the dissemination of dengue literature.

## Highlights

• Primary school teachers and guardians were main information sources on dengue.
• Children with dengue experience perceived dengue as a serious concern.
• Children with dengue experience supported dengue control and prevention.
• Poor dengue knowledge represents a challenge for school health education.

## 1 Introduction

Dengue virus (DENV) is a significant concern throughout tropical and subtropical regions where primary *Aedes* mosquito vectors are present [1]. In Thailand, dengue infection is hyperendemic [2], and incidences have fluctuated over time across its provinces [3]. In 2018, the numbers of dengue cases, reported by the national surveillance system, was highest in the 10–14 years age group, followed by the age group 5–9 years [4]. Dengue can manifest with a wide spectrum of clinical presentations, ranging from a mild non-specific febrile syndrome to severe symptoms, including plasma leakage [5]. One major risk factor of developing severe dengue is related to secondary infection with a different DENV serotype (DENV-1 to 4) from initial infection [6]. A second infection of dengue may elicit an antibody-dependent enhancement response, which aggravates vascular permeability and hemostatic disorder, leading to shock and death [7].Currently, treatment for dengue is only symptomatic, and the available vaccine does not provide equal protection against all four serotypes [8]. Thus, the primary prevention strategy relies on controlling mosquito vectors and reducing human-vector contact to reduce transmission [9].

School-based health education is a crucial tool to enhance knowledge and raise awareness of the seriousness of dengue among schoolchildren, and to transfer knowledge and practices from classrooms to homes, since, the disease is prevalent among schoolchildren, and vector control measures require continued efforts to reduce mosquito larval habitats [10]. In Thailand, a primary school-based participatory program increased children's knowledge of and participation in dengue prevention and control, resulting in a decrease in larval indices in primary schools and students' households [11]. Determination of factors associated with knowledge, attitudes, and practices (KAP) regarding dengue, is crucial for tailoring educational and behavioral interventions, and targeting subjects for participation in interventions. Previous studies have shown that people aged 17 and older, with experience of dengue, were more likely to possess more meaningful knowledge [12], more positive attitudes [13], and more effective preventive practices [14] about dengue than people without such experience. While they were experiencing the disease, patients may have sought health information or received information from healthcare providers [12]. However, few studies have assessed the impact of children's experiences with dengue on KAP, especially in Thailand.

Kanchanadit district in rural Surat Thani province in southern Thailand recorded the highest incidence of dengue in the province between 2014 and 2018, with an average of 161.8 (range 65.2–317.3) cases per 100,000 population [15]. This district adopted a larval indices surveillance system to achieve a sustainable dengue solution [16]. Surveillance activities were conducted by village health volunteers, who coordinated with primary care units. Activities involved routine household surveys to identify larval habitats, destruction of mosquito breeding habitats, and dengue death prevention campaigns. However, strategies were lacking for dengue prevention and control in primary schools. Therefore, this study assessed and compared the KAP of primary school children with and without experiences of previous dengue infection in 50 public schools throughout Kanchanadit district. This study provides useful information for enacting preventive interventions focusing on the mosquito-borne disease affecting children.

## 2 Materials and methods

### 2.1 Design and participants

A cross-sectional study was conducted in the 50 public primary schools of Kanchanadit district in Surat Thani province, approximately 651 km south of Bangkok, the capital of Thailand [17]. Primary school students between ages 8 and 13 years, who were receiving primary education (Grades 4–6) in the Thai education system [18] during the daytime between October and November 2019 were recruited. The appropriate sample size was determined for a finite population using the following formula [19]:

n=Np1−pz1−α22d2N−1+p1−pz1−α22

The minimum required sample size (*n*) was 1983 given a population (N) of 3156 [20], an expected prevalence of dengue infection in primary school children (p) of 3.6% [21], and an acceptable margin of error (d) of 0.5% at the 95% confidence level. In each primary school (stratum), children were randomly selected by proportional allocation. Students who had migrated from other districts and countries within the previous six months, or who were unable to comply with consent procedures were excluded.

### 2.2 Instruments

The KAP questionnaire was developed according to a literature review of KAP surveys retrieved from PubMed, Scopus, the Cumulative Index to Nursing & Allied Health Literature (CINAHL), and Thai-Journal Citation Index (TCI). The questionnaires were approved by three experts in the field of dengue prevention and control, and the scale-level content validity index based on the universal agreement method (S-CVI/UA) was evaluated to ensure all items in the draft tool were relevant for the study purpose. The S-CVI/UA values of the three domains (knowledge, attitudes, and practice) were 0.93, 0.96, and 0.90, respectively. An S-CVI/UA value of ≥0.8 denoted acceptable content validity [22]. The questionnaire collected (1) socio-demographic information (age, gender, education level, and parental occupation); (2) health information relating to the student's history of dengue and sources of information on dengue; (3) the knowledge domain of common symptoms, dengue vector, dengue transmission, and dengue epidemics via 10 items; (4) the attitude domain of seriousness risk and prevention via 7 items; and (5) the practice domain of methods used to reduce mosquito breeding sites and mosquito human contact via 9 items. Instrument reliability was pre-tested among 120 primary school students aged 8–13 years in Kanchanadit district; these data were not included in the final analysis. Cronbach's α was used to assess the internal consistency of the questionnaire [23]: values were 0.70 (knowledge), 0.74 (attitudes), and 0.80 (practice). Values ≥0.70 denote acceptable reliability [24].

The knowledge domain was evaluated via four multiple-choice options per item. Each correct answer scored 1 point, and each wrong answer scored 0 points. A four-point scale, with options of “strongly agree” (2 points), “agree” (1 point), “not sure” (0 points), and “disagree” (0 points) was used to identify participants' attitudes. Likewise, the practice domain used a four-point scale: “always” (3 points), “often” (2 points), “sometimes” (1 point), and “never” (0 points). The maximum score for KAP domains was 10, 14, and 27, respectively. The level of KAP were categorized as “good” or “poor” based on an 80% cut-off point [25].

### 2.3 Data collection

Data collections were done at the children's school during school hours, and at times previously agreed upon and permitted by the administration and teachers to avoid interfering with academic activities and the school routine. The 32-item questionnaire was distributed to the volunteer students who obtained written consent from their parents or guardians. A briefing concerning the procedure was provided to the students in a clear language, prior to completing the questionnaire. Volunteer students were allocated 30 min to complete the questionnaires. When testing children in this age group, issues arising from a short attention span were common [26]. To overcome attention span decay, after the first 15 min, the testing was paused, for a 10-min break that included snacks and milk, followed by another 15 min of testing [27]. Children were able to end the testing at any time. The non-response rate was 0.20%; those data were not analyzed.

### 2.4 Analysis

All completed questionnaires were double-checked and verified for completeness and consistency on the same day. The Chi-square test was used to assess the differences in distribution of a categorical variable between children with and without dengue experiences. The KAP scores of two study groups were normally distributed as revealed by the absolute values of skewness and kurtosis [28]. The Independent *t*-test was used to compare mean KAP scores between two study groups. Odds ratio (OR) with 95% confidence intervals (CI) was calculated to measure the strength of association between KAP levels (good vs. poor) and children's experience with past infection. Pearson's correlation analysis was used to describe the strength and direction of the relationships between knowledge and attitude, knowledge and practice, and attitude and practice. *P*-values <0.05 were considered to indicate statistical significance. All statistical analyses were performed using the Statistical Package for Social Sciences (SPSS; version 11.5, SPSS, Inc., Chicago, IL, USA).

## 3 Results

### 3.1 Socio-demographic characteristics and sources of information on dengue

Among the 1979 children (mean age: 10.77 years, SD: 0.93) interviewed using the structured questionnaire, 15.6% (*n* = 308) self-reported that they had been infected with dengue in the past, while 84.4% (*n* = 1671) reported no history of dengue. There were no significant differences in socio-demographic characteristics between children with and without dengue experience. Most children reported that they had heard of dengue primarily from teachers (73.6%), secondarily from parents (68.5%), and lastly, via television (57.3%). Children with experience of dengue were significantly more likely to have obtained information on dengue from their families (*P* < 0.001) and neighbors (*P* = 0.034) than children without dengue experience (see supplementary Table 1, which shows socio-demographic characteristics of participants and their sources of information on dengue).

### 3.2 Knowledge of dengue prevention

Table 1 shows that 97.5% of all children correctly answered that dengue infection is transmitted by the *Aedes* mosquito. A minority of the children (13.0%) knew that longevity of the dengue vector is generally 30–45 days, the *Aedes* mosquito usually bites people during daytime (24.5%), and its flight range is 50–100 m (49.0%). Significantly more children with dengue experience than without it, responded that severe dengue is primarily a disease affecting children (*P* = 0.016), and dengue shock can cause death (*P* = 0.019).

**Table 1:** Distribution of participants' responses to items in the dengue-related knowledge domain.

Item	Response	Number (percentage)			*P*-value
		Total *n* = 1979	Children with dengue experience, (*n* = 308)	Children without dengue experience, (*n* = 1671)	
1. *Aedes* mosquito is a vector of dengue.	Correct answer	1929 (97.5)	302 (98.1)	1627 (97.4)	0.481
	Wrong answer	50 (2.5)	6 (1.9)	44 (2.6)	
2. Female *Aedes* mosquito feeds on blood of humans.	Correct answer	1492 (75.4)	226 (73.4)	1266 (75.8)	0.372
	Wrong answer	487 (24.6)	82 (26.6)	405 (24.2)	
3. Children are at highest risk of severe dengue.	Correct answer	822 (41.5)	147 (47.7)	675 (40.4)	0.016^⁎^
	Wrong answer	1157 (58.5)	161 (52.3)	996 (59.6)	
4. Flight distances of *Aedes* mosquitoes usually range from 50 to 100 m.	Correct answer	970 (49.0)	142 (46.1)	828 (49.6)	0.266
	Wrong answer	1009 (51.0)	166 (53.9)	843 (50.4)	
5. High fever lasting 2–7 days is a common symptom of dengue.	Correct answer	1190 (60.1)	184 (59.7)	1006 (60.2)	0.879
	Wrong answer	789 (39.9)	124 (40.3)	665 (39.8)	
6. Dengue outbreaks usually coincide with the rainy season.	Correct answer	1619 (81.8)	253 (82.1)	1366 (81.7)	0.869
	Wrong answer	360 (18.2)	55 (17.9)	305 (18.3)	
7. The adult lifespan of the dengue mosquito is generally 30–45 days.	Correct answer	258 (13.0)	37 (12.0)	221 (13.2)	0.561
	Wrong answer	1721 (87.0)	271 (88.0)	1450 (86.8)	
8. Dengue mosquitoes are most likely to bite during the daytime.	Correct answer	484 (24.5)	63 (20.4)	421 (25.2)	0.075
	Wrong answer	1495 (75.5)	245 (79.6)	1250 (74.8)	
9. A common breeding site of dengue mosquito is water-holding containers around households.	Correct answer	1655 (83.6)	260 (84.4)	1395 (83.5)	0.684
	Wrong answer	324 (16.4)	48 (15.6)	276 (16.5)	
10. Dengue shock can be a leading cause of death.	Correct answer	1245 (62.9)	212 (68.8)	1033 (61.8)	0.019^⁎^
	Wrong answer	734 (37.1)	96 (31.2)	638 (38.2)	

The proportions of children answering each item were compared between groups using Chi-square test. Asterisks indicate a significant difference between groups: ^⁎^*P* < 0.05.

### 3.3 Attitudes towards dengue prevention

Table 2 summarizes children's attitudes towards dengue prevention. Most children strongly agreed (77.9%) or agreed (12.2%) that the number of dengue cases can decrease by eliminating mosquito larvae. Approximately 7% of the children disagreed that dengue is an increasingly important public health concern in Thailand. Significantly more children dengue experience than without it believed that people can die from dengue (*P* = 0.002), and that they were at risk of contracting dengue (*P* < 0.001).

**Table 2:** Distribution of participants' responses to items in the attitude domain regarding dengue prevention.

Item	Response	Number (percentage)			*P*-value
		Total *n* = 1979	Children with dengue experience (*n* = 308)	Children without dengue experience (*n* = 1671)	
1. People can die of dengue.	Strongly agree	1350 (68.2)	184 (59.7)	1166 (69.8)	0.002^⁎⁎^
	Agree	455 (23.0)	83 (27.0)	372 (22.3)	
	Not sure	121 (6.1)	28 (9.1)	93 (5.5)	
	Disagree	53 (2.7)	13 (4.2)	40 (2.4)	
2. You are at risk of getting dengue.	Strongly agree	747 (37.8)	149 (48.4)	598 (35.8)	<0.001^⁎⁎⁎^
	Agree	756 (38.2)	108 (35.0)	648 (38.8)	
	Not sure	311 (15.7)	40 (13.0)	271 (16.2)	
	Disagree	165 (8.3)	11 (3.6)	154 (9.2)	
3. Dengue can be prevented.	Strongly agree	1123 (56.7)	170 (55.2)	953 (57.0)	0.901
	Agree	470 (23.8)	78 (25.3)	392 (23.5)	
	Not sure	217 (11.0)	33 (10.7)	184 (11.0)	
	Disagree	169 (8.5)	27 (8.8)	142 (8.5)	
4. Community members have a responsibility to prevent the spread of dengue.	Strongly agree	1108 (56.0)	164 (53.3)	944 (56.5)	0.512
	Agree	559 (28.2)	94 (30.5)	465 (27.8)	
	Not sure	201 (10.2)	29 (9.4)	172 (10.3)	
	Disagree	111 (5.6)	21 (6.8)	90 (5.4)	
5. Dengue is a major public health problem in Thailand.	Strongly agree	1052 (53.1)	158 (51.3)	894 (53.5)	0.261
	Agree	544 (27.5)	78 (25.3)	466 (27.9)	
	Not sure	245 (12.4)	45 (14.6)	200 (12.0)	
	Disagree	138 (7.0)	27 (8.8)	111 (6.6)	
6. Elimination of mosquito larvae can reduce the number of dengue cases.	Strongly agree	1542 (77.9)	233 (75.6)	1309 (78.3)	0.389
	Agree	242 (12.2)	45 (14.6)	197 (11.8)	
	Not sure	108 (5.5)	14 (4.6)	94 (5.6)	
	Disagree	87 (4.4)	16 (5.2)	71 (4.3)	
7. In the future, the number of dengue cases is likely to increase in Thailand.	Strongly agree	830 (41.9)	134 (43.5)	696 (41.6)	0.453
	Agree	712 (36.0)	106 (34.4)	606 (36.3)	
	Not sure	301 (15.2)	52 (16.9)	249 (14.9)	
	Disagree	136 (6.9)	16 (5.2)	120 (7.2)	

Chi-square test. Asterisks indicate a significant difference among groups: ^⁎⁎^*P* < 0.01, ^⁎⁎⁎^*P* < 0.001.

### 3.4 Preventive practices against dengue

Table 3 shows the responses of the children regarding various preventive practices. In terms of reducing mosquito breeding sites at home, covering water-storage containers with lids was the most highly reported action by children (62.0% always, 16.8% often, and 9.8% sometimes). In terms of reducing mosquito human contact, 18.2% of the children reported they never used an electric mosquito swatter and 17.3% reported never using mosquito repellent lotion. Significantly more children with dengue experience than without it, used guppies to reduce larvae, used mosquito repellent lotion, wore long-sleeved clothing, and kept the house tidy to prevent mosquito bites (*P* = 0.005, *P* = 0.014, *P* = 0.013, and *P* = 0.031, respectively).

**Table 3:** Distribution of participants' responses to items in the practice domain regarding dengue prevention.

Item	Response	Number (percentage)			*P*-value
		Total *n* = 1979	Children with dengue experience (*n* = 308)	Children without dengue experience (*n* = 1671)	
1. Change water in small indoor containers every 7 days, e.g., flower vases	Always	695 (35.1)	121 (39.3)	574 (34.4)	0.231
	Often	683 (34.5)	107 (34.7)	576 (34.5)	
	Sometimes	317 (16.0)	44 (14.3)	273 (16.3)	
	Never	284 (14.4)	36 (11.7)	248 (14.8)	
2. Cover household water-storage containers with lids	Always	1227 (62.0)	195 (63.3)	1032 (61.8)	0.209
	Often	333 (16.8)	40 (13.0)	293 (17.5)	
	Sometimes	193 (9.8)	35 (11.4)	158 (9.5)	
	Never	226 (11.4)	38 (12.3)	188 (11.2)	
3. Use guppy fish in household water containers to consume mosquito larvae	Always	863 (43.6)	158 (51.3)	705 (42.2)	0.005^⁎⁎^
	Often	400 (20.2)	55 (17.9)	345 (20.6)	
	Sometimes	268 (13.5)	26 (8.4)	242 (14.5)	
	Never	448 (22.6)	69 (22.4)	379 (22.7)	
4. Dispose water in containers immediately when you observe mosquito larvae	Always	912 (46.1)	149 (48.4)	763 (45.7)	0.673
	Often	534 (27.0)	80 (26.0)	454 (27.2)	
	Sometimes	281 (14.2)	38 (12.3)	243 (14.5)	
	Never	252 (12.7)	41 (13.3)	211 (12.6)	
5. Dispose plastic and glass wastes serving as larval habitats	Always	831 (42.0)	138 (44.8)	693 (41.5)	0.473
	Often	497 (25.1)	68 (22.1)	429 (25.7)	
	Sometimes	314 (15.9)	46 (14.9)	268 (16.0)	
	Never	337 (17.0)	56 (18.2)	281 (16.8)	
6. Use mosquito repellent lotion	Always	738 (37.3)	121 (39.3)	617 (36.9)	0.014^⁎^
	Often	555 (28.1)	68 (22.1)	487 (29.2)	
	Sometimes	343 (17.3)	69 (22.4)	274 (16.4)	
	Never	343 (17.3)	50 (16.2)	293 (17.5)	
7. Wear long-sleeved shirts and long pants to prevent mosquito bites	Always	652 (33.0)	107 (34.7)	545 (32.6)	0.013^⁎^
	Often	372 (18.8)	74 (24.0)	298 (17.8)	
	Sometimes	309 (15.6)	35 (11.4)	274 (16.4)	
	Never	646 (32.6)	92 (29.9)	554 (33.2)	
8. Use an electric mosquito swatter	Always	672 (34.0)	109 (35.4)	563 (33.7)	0.385
	Often	541 (27.3)	93 (30.2)	448 (26.8)	
	Sometimes	405 (20.5)	56 (18.2)	349 (20.9)	
	Never	361 (18.2)	50 (16.2)	311 (18.6)	
9. Help your parents to keep the house tidy to prevent the creation of mosquito habitat	Always	1032 (52.1)	181 (58.8)	851 (50.9)	0.031
	Often	486 (24.6)	57 (18.5)	429 (25.7)	
	Sometimes	278 (14.0)	40 (13.0)	238 (14.2)	
	Never	183 (9.3)	30 (9.7)	153 (9.2)	

Chi-square test. Asterisks indicate a significant difference among groups: ^⁎^*P* < 0.05, ^⁎⁎^*P* < 0.01.

### 3.5 Overall KAP scores

Table 4 shows the mean KAP scores of the participants. The mean dengue-related knowledge score for each individual participant was 5.89 (SD: 1.69, range: 1–10), suggesting an overall 58.9% (5.89/10*100) correct rate on this knowledge test. The overall mean scores of attitude and prevention among the study participants was 9.72 (SD 2.51, range 1–14) and 17.37 (SD 5.49, range 0–27), respectively. There were no significant differences in the mean KAP scores between children with and without dengue experience (*P* > 0.05).

**Table 4:** Comparison of mean knowledge, attitude, and practice scores between children with and without dengue experience.

KAP scores	Total *n* = 1979	Children with dengue experience (*n* = 308)	Children without dengue experience (*n* = 1671)	*P*-value
Knowledge	5.89 ± 1.44	5.93 ± 1.33	5.89 ± 1.46	0.623
Attitude	9.72 ± 2.51	9.66 ± 2.59	9.73 ± 2.49	0.653
Practice	17.37 ± 5.49	17.89 ± 5.67	17.27 ± 5.45	0.079

Independent *t*-test was used for comparison KAP scores between two study groups. Data was expressed as mean ± standard deviation.

### 3.6 Association between KAP levels and children's experience with past infection

Of the children, 12.3% (244/1979*100) achieved a knowledge score of at least 80% (good knowledge), 41.6% (823/1979*100) achieved an attitude score of at least 80% (good attitude), and 25.9% (513/1979*100) achieved a preventive practice score of at least 80% (good practice). Children who had a good preventive practice were 1.34 times more likely to have a previous dengue infection than those having a poor preventive practice (OR = 1.34, 95% CI: 1.03–1.74, *P* = 0.032; Table 5). There were no statistically significant associations between experience of past infection and levels of knowledge (*P* = 0.996) and attitudes (*P* = 0.623) towards dengue prevention.

**Table 5:** Associations between levels of knowledge, attitudes, and practices and dengue experience among primary school students.

	Good level	Poor level	OR	95%CI	*P*-value
	*n* (%)	*n* (%)			
Knowledge					
With dengue experience	38 (12.3)	270 (87.7)	1.001	0.692–1.448	0.996
Without dengue experience	206 (12.3)	1465 (87.7)	1		
Attitude					
With dengue experience	132 (42.9)	176 (57.1)	1.064	0.832–1.360	0.623
Without dengue experience	691 (41.4)	980 (58.6)	1		
Practice					
With dengue experience	95 (30.8)	213 (69.2)	1.337	1.025–1.744	0.032^a^
Without dengue experience	418 (25.0)	1253 (75.0)	1		

a *P* < 0.05 was considered statistically significant. Cut-off points for good levels of knowledge, attitudes, and practices were ≥ 8, ≥11 and ≥ 22 scores, respectively. OR, odds ratio; CI, confidence interval.

### 3.7 Correlations between knowledge, attitude, and practice scores

Table 6 presents a weak positive correlation between attitudes and practices (*r* = 0.193, *P* < 0.001), while no linear relationship were seen between knowledge and attitudes (*r* = 0.054, *P* = 0.071) or knowledge and practices (*r* = 0.022, *P* = 0.325). Positive but weak linear correlations between attitudes and practices were also found in children with (*r* = 0.268, *P* < 0.001) and without dengue experience (*r* = 0.178, *P* < 0.001).

**Table 6:** Correlations between knowledge, attitude and practice scores towards dengue prevention.

Variables	Total *n* = 1979		Children with dengue experience, *n* = 308		Children without dengue experience, *n* = 1671	
	Pearson correlation	*P-*value	Pearson correlation	*P-*value	Pearson correlation	*P-*value
Knowledge & attitudes	0.054	0.071	0.003	0.955	0.064	0.090
Knowledge & practices	0.022	0.325	0.006	0.918	0.025	0.316
Attitudes & practices	0.193	<0.001^a^	0.268	<0.001^a^	0.178	<0.001^a^

a *P* < 0.001 was considered statistically significant.

## 4 Discussion

In this study, the status of previous dengue infection was obtained from self-reports. In children, initial infection with any DENV serotype ranged from asymptomatic to mild febrile illness [29], and symptoms of dengue mimicked other viral illnesses such as influenza, presenting a confusing clinical situation and challenges in identification [30]. Surprisingly, 40% of study participants did not know that the most common symptom of dengue is high fever typically lasting 2–7 days. Thus, the self-reported interpretation of previous dengue infection may have been affected by undetected symptoms, poor knowledge of dengue symptoms, and confusion between dengue symptoms and other febrile illnesses. In fact, the information on more clinical signs and symptoms of dengue e.g., bleeding (epistaxis, gum bleeding, coffee-ground vomiting, menstruation, melena), aches and pains (headache, retro-orbital pain, myalgia, arthralgia) and rash, as well as guidelines for management of suspected dengue cases should be included in school-based health education so children and their parents or guardians would know how to best manage dengue illness.

This study shows that despite living in a dengue-endemic area, most students had poor knowledge regarding dengue prevention. This is consistent with a cross-sectional study of secondary school students in Bangkok, Thailand [31] and Jazan, Saudi Arabia [32]. Most participants in this study were unfamiliar with the dengue vector's feeding time, flight distance, and lifespan. Blood-feeding facilitates mosquito vectors' transmission of DENV; thus, it is reasonable to consider how blood-feeding behavior and mosquito dispersal can be manipulated to minimize the risk of being bitten. For example, a study in rural Thailand indicated that female *Ae. aegypti* often live in or around houses where they became adults, if human hosts and oviposition sites are abundant [33]. Therefore, it is essential to apply insecticide around a dengue patient's home or community to prevent the spread of dengue. Moreover, knowing that dengue vectors actively bite during the day may encourage individuals to adopt personal protective measures, such as application of repellents.

More than 70% of the participants reported that teachers were their predominant source of information on dengue infection, followed by parents and television. In contrast, studies from Malaysia [34] and Pakistan [35] reported that the most common source of dengue knowledge among children, was television. A longitudinal study of mass media exposure regarding dengue prevention and control during between 2013 and 2015 reported a lack of effective and consistent media campaigns in Thailand, and traditional media, including television and radio, were not among the most influential sources of information on dengue [36]. Moreover, teachers in rural areas [37], often have a powerful influence on students' lives [38]. Teachers can provide nurturing and supportive relationships with students and their families that can improve learning in the classroom and beyond [39]. These results indicate that the involvement of teachers, parents, and the community may facilitate the sustainability of health interventions in primary schools.

No statistically significant association between experience of past infection and dengue-related knowledge was discovered. Previous studies reported associations between a personal history of dengue and dengue knowledge [12,34], whereas others found no association [13,40]. Studies differed in their methodology, such as determination of dengue knowledge, demographics of the participants, and use of cut-off points for determining “poor” and “good” levels. Based on studies of personal and demographic factors affecting dengue-related knowledge, children's knowledge may be associated with their parents' level of education [41]. Parents with higher levels of education may facilitate access to information when their child encounters the disease and may have the ability to understand complex information from healthcare staff when their child receives treatment. Such parents are also more likely to transfer this information to their children [41]. Moreover, acquisition of basic dengue knowledge in primary schools requires teacher competency [42]. In rural southern Thailand, health education for dengue prevention and control is provided in primary schools; however, this educational program receives low priority, strategies and materials are inconsistently reviewed, and teachers lack training to address dengue problems in their schools [43].

Regarding attitudes towards dengue prevention, children with dengue experiences were significantly more likely than those without such experiences to think that people can die of dengue, and they were at risk of contracting dengue. Thus, children with dengue experience perceived dengue as a serious concern. However, there was no significant association between dengue experience and attitudes towards the disease, consistent with a previous study of university students in Malaysia [44]. In contrast, other studies of healthy adults in Indonesia [13] and Columbia [40] reported that dengue experience is associated with attitudes towards dengue prevention. Changing attitudes towards health remain an issue; although school-based health education was utilized, children's attitudes towards the disease did not improve [34,45].

Dengue experience was significantly and positively associated with dengue prevention practices. This was especially evident for practices regarding the use of guppies and mosquito repellent lotion, wearing long-sleeved clothing, and maintaining a clean home. This shows that children with dengue experience supported dengue prevention. Study findings are consistent with a study of secondary school students in Malaysia, which reported that dengue history affected the level of prevention practices [34], but contrast with a study of healthy adults in Malaysia [12]. Because primary school students are not yet permitted to make their own decisions with full independence, implementation of dengue preventive measures by children requires support, direction, and supervision by parents, guardians, or teachers. Thus, one reason dengue experience affected preventive practices in this study may be that parents of children who had dengue had experienced the seriousness of the disease and might have guided their child towards personal protection and environmental control of the vector.

No correlation was observed between knowledge and attitude, or between knowledge and practices among primary school students. This suggests that prior knowledge of dengue was ineffective at improving the children's attitudes and practices. Knowledge of dengue prevention is advantageous but does not guarantee the adoption of preventive practices. However, a positive significant correlation between attitudes and practices was found among the participants. This suggests that attitudes affected the preventive practices of the participants. That is, perceptions of low risk may minimize incentives to act against dengue [12]. Therefore, educational interventions must highlight the risk of contracting the disease, regardless of the child's prior dengue experience, to create awareness of the dengue threat.

There are some limitations of the present study. All information was acquired via self-report, and whether the reported practices accorded with actual practices could not be verified. A social-desirability bias might have existed in responses to questions within the attitude and practice domains. Additionally, the cross-sectional design does not permit the interpretation of causal relationships among factors. In contrast, an important strength of this study is that participants were recruited from 50 primary schools across the district, suggesting results can be applied to community settings in general. The eligibility criteria further strengthened the quality of the findings.

## 5 Conclusions

This study provides current data on baseline KAP regarding dengue prevention among primary school students in a rural area in southern Thailand. The findings may help identify intervention groups for school-based education programs, and the design and development of intervention programs to protect the health of vulnerable groups in primary school.

This study suggests that relatively good attitudes and practices were present regarding dengue prevention, although most children possessed a poor level of dengue-related knowledge. Therefore, there is a need for appropriate dengue knowledge to be included in school curricula, emphasizing not only preventive measures but also clinical signs/symptoms and initial management to raise a comprehensive understanding and awareness of the disease. In particular, the mobilization of children with dengue experience will be important in this context as such children were supportive of dengue prevention and control. Moreover, primary school teachers and parents are common sources of dengue-related information, rather than mass media. Accordingly, school-based interventions should rely on the involvement of family to support and improve the learning, development, and health of children.

## Authors' contributions

The conception and design of the study, and acquisition of data were performed by C.S., B.S., T.S., N.T and S.M. Analysis and interpretation of data were executed by S.M. and C.S. Writing an initial draft, as well as revising and editing drafts were executed by S.M. All authors extensively revised and approved the final version of the manuscript.

## Ethics approval and consent to participate

The study protocol followed the principles of the Declaration of Helsinki and was approved by the Human Research Ethics Committee of Walailak University (WUEC-19-145-01; dated 20/09/2019). Permission to collect the data was obtained from the directors of primary schools. Informed consent was obtained from all primary students and their parent or legally authorized representative, after the purpose of the study was explained to them. They were also informed that their participation in the study was voluntary, and they may withdraw from participation any time. Each participant's questionnaire was anonymized for protection and privacy.

## Funding

This study was supported by the Community Public Health Program of the School of Public Health, 10.13039/501100010034Walailak University. The funder of the study had no role in study design, data collection, data analysis, data interpretation, writing of the manuscript, or the decision to submit for publication.

## Declaration of Competing Interest

The authors declare that they have no competing interests.

## Appendix A Supplementary data

Supplementary Table 1 shows socio-demographic characteristics of participants and their sources of information on dengue. Image 1
