# Supplementary material for: Knowledge, attitudes, and practices towards dengue prevention among primary school children with and without experience of previous dengue infection in southern Thailand
Source: One Health. 2021 Jun 7;13:100275. doi: 10.1016/j.onehlt.2021.100275 (PMC8203813; doi:10.1016/j.onehlt.2021.100275)
Supplement: Supplementary file 1 — Supplementary Table 1 shows socio-demographic characteristics of participants and their sources of information on dengue. [file mmc1.docx]

**Supplementary Table 1.** Socio-demographic characteristics and information sources regarding dengue of study participants.

| Variable | Total  *n* (%) | Children with dengue experience, *n* (%) | Children without dengue experience, *n* (%) | *P-*value |
| --- | --- | --- | --- | --- |
| Number | 1,979 | 308 | 1,671 |  |
| Gender |  |  |  |  |
| Male | 859 (43.4) | 143 (46.4) | 716 (42.8) | 0.244 |
| Female | 1,120 (56.7) | 165 (53.6) | 955 (57.2) |  |
| Parental occupation |  |  |  |  |
| Agricultural worker | 764 (38.6) | 118 (38.3) | 646 (38.7) | 0.905 |
| Freelancer | 746 (37.7) | 115 (37.3) | 631 (37.8) |  |
| Entrepreneur | 271 (13.7) | 44 (14.3) | 227 (13.6) |  |
| Private employee | 76 (3.8) | 12 (3.9) | 64 (3.8) |  |
| Government employee | 43 (2.2) | 9 (2.9) | 34 (2.0) |  |
| Unemployed worker | 79 (4.0) | 10 (3.3) | 69 (4.1) |  |
| Source of information regarding dengue |  |  |  |  |
| Teacher | 1,456 (73.6) | 219 (71.1) | 1,237 (74.0) | 0.285 |
| Parents | 1,356 (68.5) | 250 (81.2) | 1,106 (66.2) | < 0.001^***^ |
| Television | 1,134 (57.3) | 176 (57.1) | 958 (57.3) | 0.951 |
| Website | 723 (36.5) | 110 (35.7) | 613 (36.7) | 0.745 |
| Friend | 694 (35.1) | 106 (34.4) | 588 (35.2) | 0.794 |
| Neighbor | 624 (31.5) | 113 (36.7) | 511 (30.6) | 0.034^*^ |
| Health worker | 523 (26.4) | 73 (23.7) | 450 (26.9) | 0.238 |
| Book | 521 (26.3) | 81 (26.3) | 440 (26.3) | 0.990 |
| Community leader | 324 (16.4) | 58 (18.8) | 266 (15.9) | 0.204 |
| Newspaper | 277 (14.0) | 37 (12.0) | 240 (14.4) | 0.275 |
| Radio | 249 (12.6) | 33 (10.7) | 216 (12.9) | 0.282 |

Children were categorized based on self-reported history of dengue. Chi-square test was used to compare characteristics between children with and without history of dengue. Asterisks indicate a significant difference between groups: ^*^*P* < 0.05, ^***^*P* < 0.001.
